# Effect of Sugar 2′,4′-Modifications on Gene Silencing Activity of siRNA Duplexes

**DOI:** 10.1089/nat.2019.0792

**Published:** 2019-08-06

**Authors:** Elise Malek-Adamian, Johans Fakhoury, Amy E. Arnold, Saúl Martínez-Montero, Molly S. Shoichet, Masad J. Damha

**Affiliations:** ^1^ Department of Chemistry, McGill University, Montreal, Canada.; ^2^ Department of Chemical Engineering and Applied Chemistry, University of Toronto, Toronto, Canada.; ^3^ Institute of Biomaterials and Biomedical Engineering, University of Toronto, Toronto, Canada.; ^4^ Department of Chemistry, University of Toronto, Toronto, Canada.

**Keywords:** ribose-modified siRNA, RNAi, oligonucleotide synthesis

## Abstract

In this study, we explore the effect of a library of 2′-, 4′-, and 2′,4′-modified uridine nucleosides and their impact on silencing firefly luciferase and on down-regulated in renal cell carcinoma (DRR) gene targets. The modifications studied were 2′-F-ribose, 2′-F-arabinose, 2′-OMe-ribose, 2′-F,4′-OMe-ribose, 2′-F,4′-OMe-arabinose, and 2′-OMe,4′-F-ribose. We found that 2′,4′-modifications are well tolerated within A-form RNA duplexes, leading to virtually no change in melting temperature as assessed by UV thermal melting. The impact of the dual (2′,4′) modification was assessed by comparing gene silencing ability to 2′- or 4′- (singly) modified siRNA counterparts. siRNAs with (2′,4′)-modified overhangs generally outperformed the native siRNA as well as siRNAs with a 2′- or 4′-modified overhang, suggesting that 2′,4′-modified nucleotides interact favorably with Argonaute protein's PAZ domain. Among the most active siRNAs were those with 2′-F,4′-OMe-ribose or 2′-F,4′-OMe-arabinose at the overhangs. When modifications were placed at *both* overhangs and internal positions, a duplex with the 2′-F (internal) and 2′-F,4′-OMe (overhang) combination was found to be the most potent, followed by the duplex with 2′-OMe (internal) and 2′,4′-diOMe (overhang) modifications. Given the nuclease resistance exhibited by 2′,4′-modified siRNAs, particularly when the modification is placed at or near the overhangs, these findings may allow the creation of superior siRNAs for therapy.

## Introduction

Exploiting the therapeutic potential of RNAi has been fueled with the recent approval of Patisiran, the first small interfering RNA (siRNA)-based drug approved for the treatment of hereditary transthyretin amyloidosis [1]. This siRNA comprises unmodified and 2′-OMe pyrimidine nucleotides that require encapsulation in a lipid-based nanoparticle for efficient delivery to the target organ [1,2]. The pursuit of novel chemical modifications that improve the biochemical (nuclease resistance, potency) and biophysical properties of oligonucleotides continues to be an important endeavor toward the development of more effective siRNA drugs that do not require a lipid-complex formulation. In this regard, although the 2′-F modification is widely introduced into siRNAs, it provides minimal stabilization toward nuclease degradation. Modifications that retain some of the properties of 2′-F nucleotides while increasing nuclease resistance are desired.

**FIG. 1.**
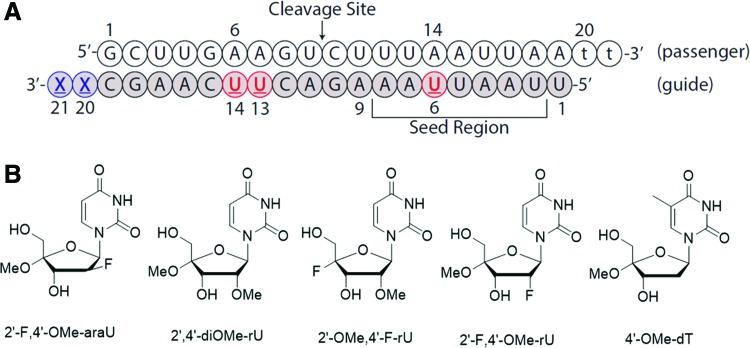
**(A)** Sequence of siRNA targeting luciferase mRNA. Modified nucleotides were introduced at positions 6, 13, and 14 and 3′-overhang of the antisense (guide) strand; **(B)** 2′,4′-modified nucleosides in this study. Color images are available online.

In a collaborative study, we reported recently on the synthesis, conformational analysis, and gene silencing activity of 2′-F,4′-OMe modified siRNAs in cultured cells, and showed that they promote efficient gene silencing [3]. More recently, our laboratory reported on the synthesis and hybridization properties of duplexes containing a variety of other 2′,4′-modified uridine nucleosides, for example, 2′,4′-di-OMe, 2′-OMe,4′-F, and ara-2′-F, 4′-OMe uridines [4]. As previously found with 2′-F, 4′-OMe uridine, this new library of uridines exhibited primarily a C3′-*endo* (“North”) sugar conformation [5] and their incorporation into siRNAs led to virtually no change in duplex melting temperature [4,6]. This property, together with the observation that C2′/C4′ modifications impart much desirable resistance toward endo/exo nuclease degradation [3,7,8], prompted us to evaluate whether these analogues are compatible with the RNA-induced silencing complex (RISC) and able to regulate gene expression through the RNAi pathway. Herein we report studies that assess the gene silencing efficiency of C2′/C4′ modified siRNA duplexes (Fig. 1). Modifications were first introduced at either internal (positions 6, 13, and 14) or terminal positions (positions 20 and 21) of the guide strand; the most potent modifications were then combined to produce siRNAs with modifications at both overhangs and internal positions. Of the various modifications tested, duplexes with a C2′-F/OMe and C4′-OMe modified ribose sugar at the 3′-overhangs were among the most potent.

## Materials and Methods

### Oligonucleotide synthesis

All 2′,4′-modified phosphoramidites and oligonucleotides were synthesized as previously reported [4]. The 4′-OMe-dT phosphoramidite was synthesized from a C4′-C5′ alkene dT precursor according to Liboska *et al.* [9] (4′-OMe-dT P31 NMR spectrum Section in Supplementary Data). Oligonucleotides were purified by ion exchange high performance liquid chromatography (HPLC) (1 M lithium perchlorate buffer) or by polyacrylamide gel electrophoresis (PAGE) (22% acrylamide), and desalted as previously described [3]. All oligonucleotides were characterized by electrospray ionization (ESI) or quadrupole time-of-flight mass spectrometry (QTOF) mass spectrometry (Supplementary Table S1 and Supplementary Data).

### Thermal denaturation studies

Complementary sequences were combined in equimolar amounts (1.5 nmol), dried, and diluted in a buffer containing 10 mM sodium phosphate (pH 7.2) with 100 mM NaCl and 0.1 mM EDTA (1 mL). Each solution was then transferred into cuvettes in a Varian UV spectrophotometer. Each sample was heated to 93°C and then cooled to 15°C at a rate of 0.5°C/min. The change in absorbance at 260 nm was then monitored upon heating from 15°C to 93°C. The melting temperatures were calculated from the first derivative of the melting curves (local maxima at dy/dt = 0).

### Luciferase assays

HeLa cells stably expressing Luciferase were counted and seeded at a density of 7,500 cells/well in a 96-well plate. Cells were then left to recover for 24 h at 37°C with 5% CO_2_. Subsequently, cells were washed once with serum-free Dulbecco's modified Eagle's medium (DMEM) and then 80 μL of serum-free DMEM media was added. siRNA and control nucleic acid preparations were prepared by heating at 93°C in a heat block in 1 × siRNA buffer (instructions for preparation by Dharmacon) and were allowed to anneal slowly followed by overnight cooling at 5°C in a fridge. siRNA duplexes were then diluted up to 20 μL with serum-free media and transfection reagent (Oligofectamine; Invitrogen) and added to the appropriate well (for a total of 100 μL) at increasing concentrations (0.16, 0.8, 4, 20, and 100 nM). Cells were incubated overnight (for a total of 24 h post-siRNA addition). Then 50 μL of ONE-Glo luciferin reagent (Promega) was added to each well and luminescence was measured and normalized to protein levels using a Biotek Synergy HT plate reader. Data were acquired with the Gen5 software suite and were manipulated and plotted using Graphpad Prism software suite.

### DsRed DRR cell transfection

This protocol was adapted from Anzahaee *et al.* [10]. In brief, DsRed DRR cells were seeded in a 6- or 24-well plate such that they would reach 60%–80% confluency at the time of transfection. siRNAs were complexed to Lipofectamine RNAiMax (Thermo 13778075) for 5 min in Opti-MEM media (Thermo 31985062) according to the manufacturer's reagent protocol and added to the cells in DMEM media (Gibco 11995605) for a final DMEM:Opti-MEM ratio of 1.5:0.5 and desired siRNA concentration. At 24 h, additional serum-containing DMEM media was added. After a total of 72 h of incubation at 37°C, cells were collected and lysed using 0.1% NP-40 (Fluka 74385). Protein expression was assessed through western blot analysis of DRR with alpha-tubulin as a loading control.

### Cell viability assay

Cytotoxicity of tested nucleosides was assayed by measuring cell viability using the Cell Titer Blue Assay (Promega). In brief, HeLa cells were seeded at 5,000 cells/well in a 96-well plate. The following day, samples were prepared at stock concentrations starting at 1 M in autoclaved H_2_O, and were serially diluted 10-fold down to 1 μM. Ten microliters of each stock solution was then added per well at the desired concentration to be incubated with HeLa cells for 24 h. Incubations were performed in triplicate for each concentration. After overnight incubation, 20 μL of Cell-Titer Blue reagent was added per well and sample fluorescence was measured using a Biotek Synergy Plate Reader (excitation = 560 nm and emission = 590 nm) after 1 h following addition. Analysis was performed using Graphpad Prism software and samples were normalized to the buffer control.

## Results and Discussion

### siRNAs with internal 2′/4′-modifications in the guide strand

Melting temperature of internally modified duplexes were studied first using a model siRNA duplex targeting firefly luciferase as previously studied [11]. This model siRNA was chosen due to its modification pattern that allocates a modified nucleotide in the sensitive seed region (position 6) and allows exploration of the impact of two centrally located and consecutive modified nucleotides (positions 13 and 14). Each duplex was analyzed by UV thermal melting to assess how these modifications affected duplex formation. In all cases, no distortions in duplex formation were noted during the melting-annealing cycle (UV thermal melting section in Supplementary Data). Relative to the native siRNA, *T*_m_ values of duplexes increased upon incorporation of modified ribouridines and 4′-OMe-dT (+1°C/nt to 2°C/nt), whereas a slight decrease (*ca.* −0.3°C/nt to 0.7°C/nt) was observed for the arabinose modifications (Table 1; Supplementary Figs. S1–S3; Supplementary Data). As previously observed, duplexes with 2′,4′ modifications were more stable than the unmodified duplexes (+0.8°C/nt to 1.4°C/nt), although they were not as stable as duplexes with 2′-modifications at the same positions [3,4,11].

**Table 1.** Melting Temperature of Luciferase siRNA Duplexes with Internal Modifications at Positions 6, 13, and 14 in the Guide Strand

*Guide strand sequence*			
5′-UUA AU**U** AAA GAC **UU**C AAG C(dGG)-3′			
**U**			
2′R	4′R	*T*_m_	Δ*T*_m_/nt
OH	H	58.1	—
F	H	64.1	+2.0
F	OMe	62.4	+1.4
OMe	H	61.2	+1.0
OMe	F	60.5	+0.8
OMe	OMe	60.4	+0.8
araF	H	56.0	−0.7
araF	OMe	57.3	−0.3
H	OMe	62.4	+1.4

RNA target is a complementary unmodified passenger strand; *bold underlined* residue refers to a 2′,4′-modified uridine nucleotide; melting curves are shown in Supplementary Fig. S1.

**FIG. 2.**
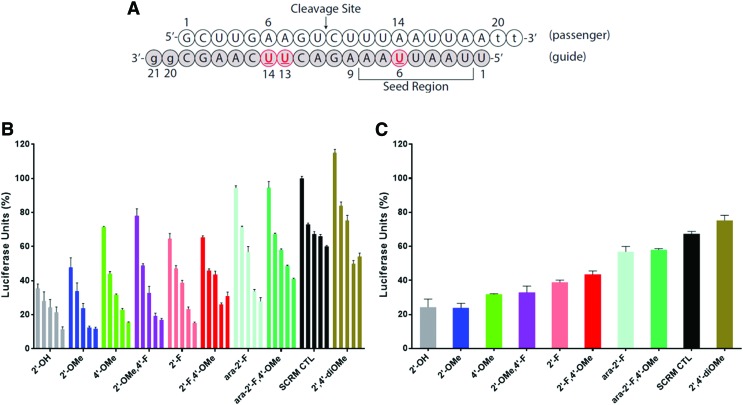
Luciferase assay results of siRNA with internal 2′,4′ modifications at positions 6, 13, and 14 of the guide strand. **(A)** Sequence design of the siRNA. Locations of modified nucleotides are *underlined*. **(B)** Activity of siRNA duplexes. The concentrations used (organized *left* to *right* are 0.16, 0.8, 4, 20, and 100 nM, respectively). **(C)** Relative gene silencing activity of duplexes at 4 nM concentration. 4′-OMe refers to the 4′-OMe-dT modification. SCRM CTL is the scramble negative control. Color images are available online.

Duplexes were transfected into HeLa cells stably expressing firefly luciferase, and the reduction of firefly luciferase protein levels was evaluated in a dose–response manner (Fig. 2). The best activity was granted by the 2′-OMe-rU modification, performing similarly to rU. As previously observed with 2′,4′-diF-rU [11], siRNAs with 2′-F,4′-OMe-rU and 2′-OMe,4′-F-rU modifications also conferred excellent activity. By contrast, decreased activity was observed with 2′,4′-diOMe-rU and 2′-F,4′-OMe-araU modifications, in line with observed results when incorporating LNA at these positions [11]. The negative impact of 2′,4′-diOMe-rU on gene silencing was not expected given the excellent activities of siRNAs with 2′-OMe-rU or 4′-OMe-dT at the same positions. This may reflect possible disruption of hAGO2-RNA interactions caused by the bulkier 2′,4′-diOMe-rU residues [6,12–14].

### siRNAs with 2′/4′-modifications at the 3′-overhang of the guide strand

**FIG. 3.**
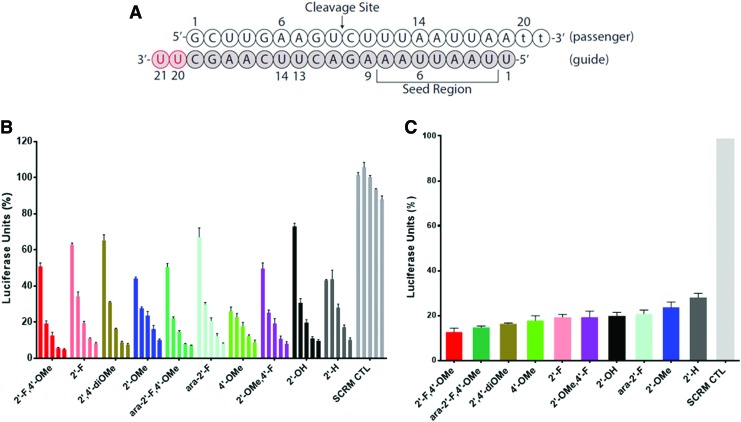
Luciferase assay of duplexes with indicated modifications at overhang positions. **(A)** Sequence of the siRNA; **(B)** activity of siRNAs modified at positions 20 and 21 of the guide strand. The concentrations used are 0.16, 0.8, 4, 20, and 100 nM (organized *left* to *right*), respectively. **(C)** Relative gene silencing activity of duplexes at 4 nM concentration. Sequence of the native siRNA is shown in Fig. 2 and contains a dGG 3′-overhang. 4′-OMe refers to the 4′-OMe-dT modification. SCRM CTL is the scramble negative control. Color images are available online.

Structural studies by Patel and coworkers revealed that the PAZ domain from human Argonaute serves as a binding module and anchoring site for the 3′ end of guide RNA, requiring an essential 2-nt, single-stranded segment [15]. To study the potential interaction of our 2′,4′-modifications with the PAZ domain, they were introduced at the 2-nt 3′-overhangs of several siRNA duplexes (positions 20 and 21 of the guide strand). Their gene silencing activity was measured at a range of concentrations from 0.16, 0.8, 4, 20, to 100 nM, and depicted in Fig. 3.

The slightly better activity of ara-2′-F versus 2′-H had previously been reported by Dowler *et al.*, and is fully consistent with the present results [16]. Regarding the 2′,4′-modified siRNAs, the instructive findings were that overhangs with C4′-α-OMe substituted furanoses provided the most active duplexes across the series. This suggests that the hydrophobic pocket of the PAZ domain interacts favorably with C4′-*O*-methyl (and even C4′-fluorine) substituents. For example, the following trends in activities were observed: 2′-F-4′-OMe >2′-F; also, ara-2′-F-4′-OMe > ara-2′-F; furthermore, 2′,4′-diOMe >2′-OMe; also ara-2′-F-4′-OMe > ara-2′-F. Further contribution to the activity of these duplexes may arise from the enhanced nuclease stability provided by the C4′-substituent, which hinders hydrolysis of the vicinal 3′,5′-phosphodiester linkage [3].

### siRNAs combining internal and overhang 2′/4′-modifications

**FIG. 4.**
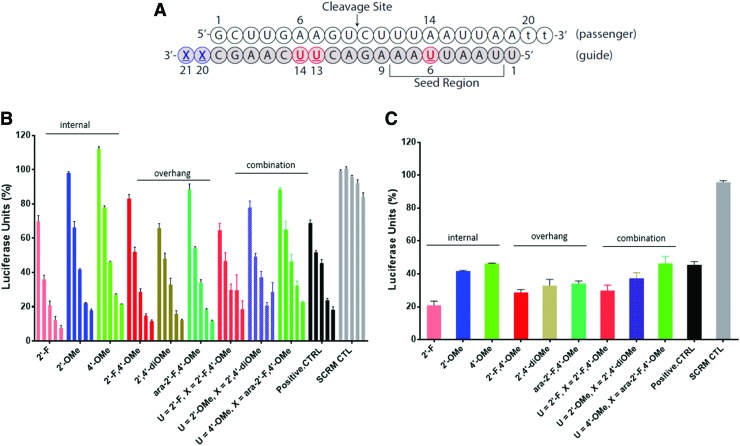
Luciferase assay of duplexes modified at positions 6, 13, 14, 20, and 21. **(A)** Sequence of the siRNA; **(B)** activity of modified duplexes. The concentrations used are 0.16, 0.8, 4, 20, and 100 nM (*left* to *right*), respectively; **(C)** relative gene silencing activity of duplexes at 4 nM concentration. Sequence of the native siRNA is shown in Fig. 2 and contains a dGG 3′-overhang. 4′-OMe refers to the 4′-OMe-dT modification. SCRM CTL is the scramble negative control. Color images are available online.

Next, we sought to combine some of the best performing internal modifications (modifications at positions 6, 13, and 14) with the best performing overhang modifications (modifications at positions 20 and 21) with the aim of maximizing chemical modification without compromising activity (Table 2). The sequence and melting temperatures of the duplexes prepared are provided in Table 2, and their activity summarized in Fig. 4. For comparison, the *T*_m_ and gene silencing activity of these duplexes were compared with siRNAs containing modifications at overhangs or internal positions.

**Table 2.** Melting Temperature of Luciferase siRNA Duplexes with a Modified Guide Strand

*Guide strand sequence*				
5′-UUA AU**U** AAA GAC **UU**C AAG C*UU*-3′				
**U**	*U*	*T*_m_	*ΔT*_m_	*ΔT*_m_/nt
2′OH	2′H	58.1	—	—
2′OH	2′F-4′OMe	62.4	+4.3	+2.2
2′F	2′OH	64.1	+6.0	+2.0
2′F	2′F-4′OMe	64.4	+6.3	+1.3
2′OH	2′,4′-diOMe	62.4	+4.3	+2.2
2′OMe	2′OH	61.2	+3.1	+1.0
2′OMe	2′,4′-diOMe	64.4	+6.3	+1.3
2′OH	ara-2′F-4′OMe	62.4	+4.3	+2.2
4′OMeT	2′OH	62.4	+6.3	+1.4
4′OMeT	ara-2′F-4′OMe	65.4	+7.3	+1.5

RNA target is a complementary unmodified passenger strand; melting curves are shown in Supplementary Figs. S1–S3. The guide strand of the unmodified siRNA has a d(GG) 3′-overhang, that is, *UU* = d(GG).

UV experiments were performed to verify if the additional modifications affected the melting temperature of the duplexes (Table 2; Supplementary Figs. S1–S3). Interestingly, the *T*_m_ of duplexes with combined modifications at positions 6, 13, 14, 20, and 21 displayed similar or slightly higher values compared with duplexes with internal modification at positions 6, 13, and 14 (Δ*T*_m_ = +0.3°C to 3.0°C). It is also interesting to note that the modified rPy overhangs tested at positions 20 and 21, namely 2′-F-4′-OMe-rU, 2′,4′-diOMe-rU and 2′-F-4′-OMe-araU all provided significant increases in *T*_m_ (+2.2°C/nt) relative to the duplex with a native rPu (dGG) overhang. Although the origin for this difference cannot be reconciled with the data available, the larger stabilizing effect of the modified rPy “dangling” ends may be attributed to an overall more cross-stacking of the modified residues, which better conform to A-form structure of the siRNA duplex [17]. In other words, the propensity of the unstacked dangling nucleotide to a stacked geometry should be easier for a nucleotide preorganized in the A-like C3′-*endo* conformation (such as 2′,4′-modified rU) relative to a more flexible deoxynucleotide (e.g., dGG).

Regarding gene silencing activity, the combinations of modifications afforded siRNAs with potencies similar to those seen for siRNAs with only overhang or only internal guide strand modifications. An exception is the siRNA with internal 2′-F units that exhibited greater activity relative to siRNAs with only overhang (2′-F,4′-OMe) or combined internal (2′-F)/overhang (2′-F,4′-OMe) modifications. Nevertheless, a desired level of activity can be achieved with the combined analogues while maintaining a high level of modification necessary for nuclease protection and immune system evasion.

### Activity of the combination of best internal and overhang motifs against DRR gene

**FIG. 5.**
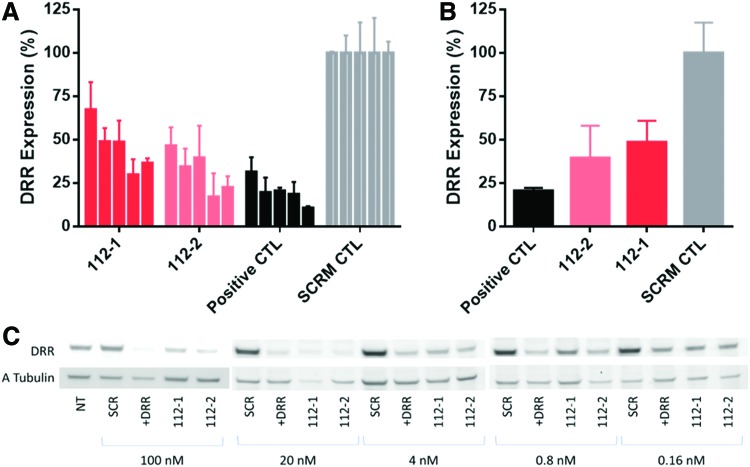
DRR knockdown after transfection of siRNAs with overhang or combined internal and overhang modifications in DsRed DRR cells. **(A)** Assay results grouped by modification type. **(B)** Relative gene silencing activity of duplexes at 4 nM concentration. **(C)** Gel image of the western blot analysis grouped by concentration. SCRM CTL is the scramble negative control. DDR, down-regulated in renal cell carcinoma. Color images are available online.

To validate the generality of our observations, we designed a new set of siRNAs targeting the downregulated in renal (DRR) cell carcinoma gene as previously reported [18,19]. The DRR protein contributes to the aggressive and invasive nature of glioblastoma stem cells by cross-linking microtubules and actin at focal adhesions and activating downstream pathways of protein kinase B [18]. Knockdown of the DRR gene is a promising therapeutic strategy for decreasing the invasion of brain cancer stem cells [19,20]. One duplex contained 2′-F-4′-OMe-rU residues at the 2-nt 3′-overhang of the guide strand; the other contained three additional 2′-F-rU residues at internal positions. Their sequences are shown in Table 3, along with that of the unmodified siRNA control. DsRed DRR cells were transfected with modified and control siRNA duplexes. All siRNA duplexes targeting the DRR gene provided significant knockdown compared with the scrambled control; the DRR positive CTRL duplex was the most active, followed by the siRNA duplex with modified overhangs (Fig. 5). The least active was the most modified (112–2); however, it still exhibited excellent activity (IC_50_ *ca.* 4 nM) under the conditions tested.

**Table 3.** Sequences of Down-Regulated in Renal Cell Carcinoma (DRR) siRNAs

*Duplex ID*	*Sequence*
DRR CTRL	5′ r(UUC UUG AUG AGC UGG UUC CUU) 3′
	3′ r(UUA AGA ACU ACU CGA CCA AGG) 5′
112-1	5′ r(UUC U**U**G AUG AGC **U**GG **U**UC C**XX**) 3′
	3′ r(UUA AGA ACU ACU CGA CCA AGG) 5′
112-2	5′ r(UUC UUG AUG AGC UGG UUC C**XX**) 3′
	3′ r(UUA AGA ACU ACU CGA CCA AGG) 5′

Top and bottom strands are the guide and passenger strands, respectively.

**U** = 2′-F-rU; **X** = 2′-F,4′-OMe-rU.

### Metabolic activity of HeLa cells upon treatment with excess modified nucleosides

**FIG. 6.**
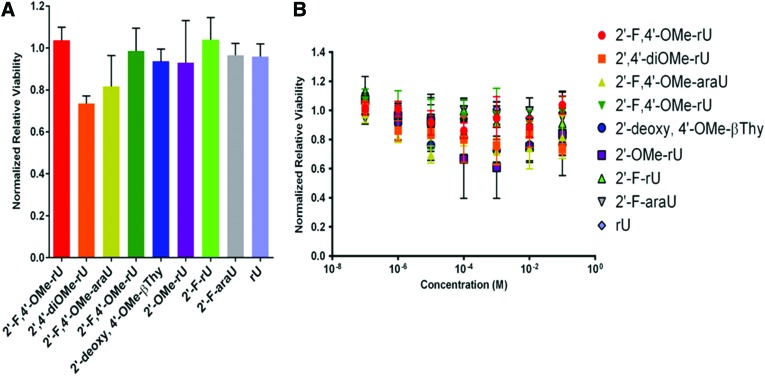
Cell viability assay results. **(A)** Normalized cell viability at highest tested concentration (0.1 M). **(B)** Normalized cell viability at range of concentrations tested. Color images are available online.

A common concern with chemically modified nucleotides is that if they were to be excised by the strand by endogenous nucleases, especially at vulnerable positions for cleavage such as at the 3′-end, they could potentially cause toxicity [21]. We addressed this concern by performing a cell viability assay of each nucleoside in this tested library at concentrations up to 0.1 M with a cell viability assay in HeLa cells, and none other than 2′,4′-diOMe-rU was shown to reduce significantly cell viability at this concentration (Fig. 6). It is important to note that 0.1 M also far exceeds physiological conditions within cells, where dUTP in mammalian cells normally hovers at *ca.* 0.5 mM [22]. Indeed, it was also recently shown that 2′-F nucleotide modifications do not cause significant toxicity in cells [23]. As such, we expect that these chemically modified nucleosides, if released during siRNA hydrolysis by nucleases and phosphatases, should not be toxic to human cells.

## Conclusions

Rapid progress has been made toward RNAi-based therapy by rationally optimizing chemically modified siRNAs for high specificity and potent gene silencing. In this study, we analyzed the effect of novel 2′,4′-modifications at different positions in the antisense strand of an siRNA targeting the firefly luciferase and DRR genes. We found that 4′-OMe or 4′-F modifications are effective at inducing gene knockdown, especially when placed at the 3′-overhang of the guide strand. We hypothesize that favorable interaction with the PAZ domain of hAGO2 and enhanced nuclease stability provided by these modifications both contribute to the improved activity of these modified siRNAs. This and other recent studies on 2′,4′-modified oligonucleotides by Egli, Manoharan and our group [3,4,6,11,24] expands the toolbox of desirable chemical modifications for gene silencing applications and opens the avenue for further studies of these chemical modifications against other gene targets.

## Supplementary Material

Supplemental data
