# Supplementary material for: Effect of Sugar 2′,4′-Modifications on Gene Silencing Activity of siRNA Duplexes
Source: Nucleic Acid Ther. 2019 Aug 6;29(4):187–94. doi: 10.1089/nat.2019.0792 (PMC6686699; doi:10.1089/nat.2019.0792)
Supplement: Supplemental data [file Supp_Data.pdf]

Supplementary Information

Effect of sugar 2',4'-modifications on gene silencing activity of small interfering RNA duplexes

Elise Malek-Adamian[1], Johans Fakhoury[1], A. Ellie Arnold[3], Saúl Martínez-Montero[4], Molly S. Shoichet[2,3,4], and Masad J. Damha[1]*

[1]Department of Chemistry, McGill University, 801 Sherbrooke Street W., Montreal, QC, H3A 0B8, Canada
[2]Department of Chemistry, University of Toronto, 80 St George Street, Toronto, ON, M5S 3H6, Canada
[3]Department of Chemical Engineering and Applied Chemistry, University of Toronto, 200 College Street, Toronto, ON, M5S 3E5, Canada
[4]Institute of Biomaterials and Biomedical Engineering, University of Toronto, 164 College Street, Toronto, ON, M5S 3G9, Canada

* Correspondence to: Email address masad.damha@mcgill.ca

Contents

S1. UV Thermal Melting 1
S2. Mass Analysis of Oligonucleotides 3
S3. 4'-OMe-dT P31 NMR Spectrum 6

S1. UV Thermal Melting

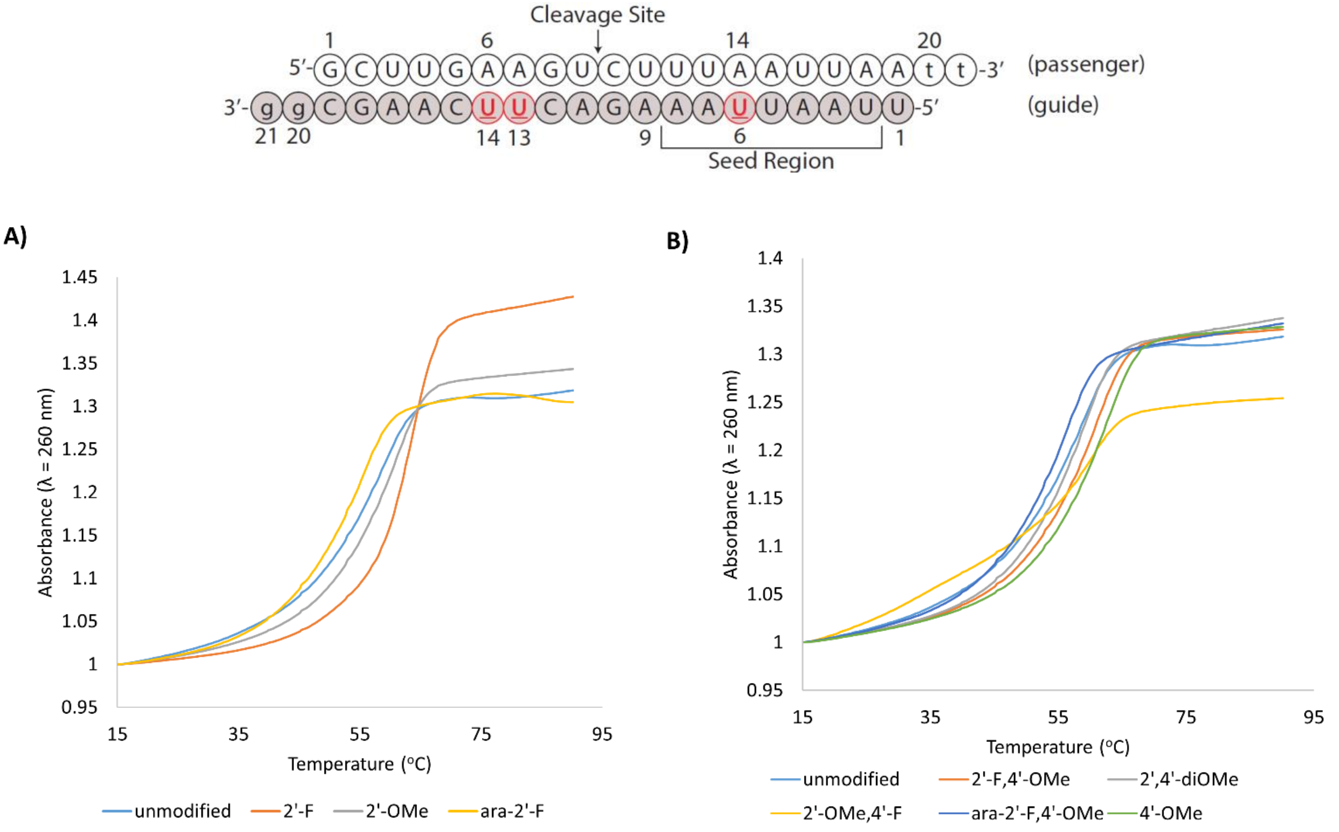

Figure S1. UV thermal melting of siRNA duplexes with modified guide strands at positions 6, 13, and 14. **A)** siRNAs with 2’-modifications. **B)** siRNAs with 2’,4’-modifications.

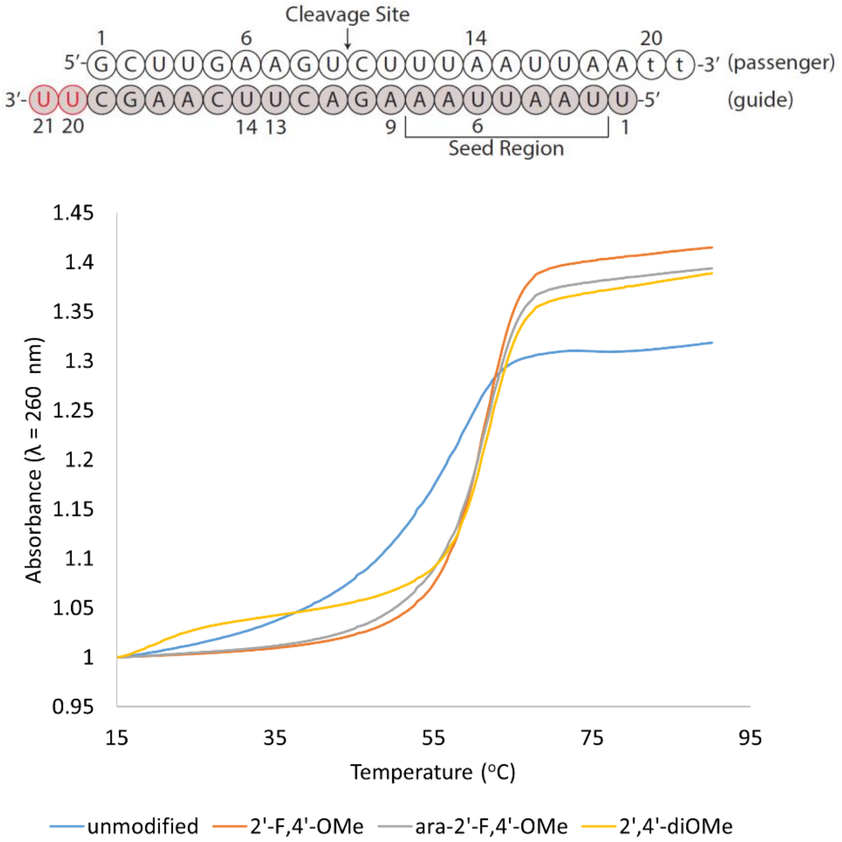

Figure S2. UV thermal melting of siRNA duplexes modified at positions 20 and 21 in the guide strand.

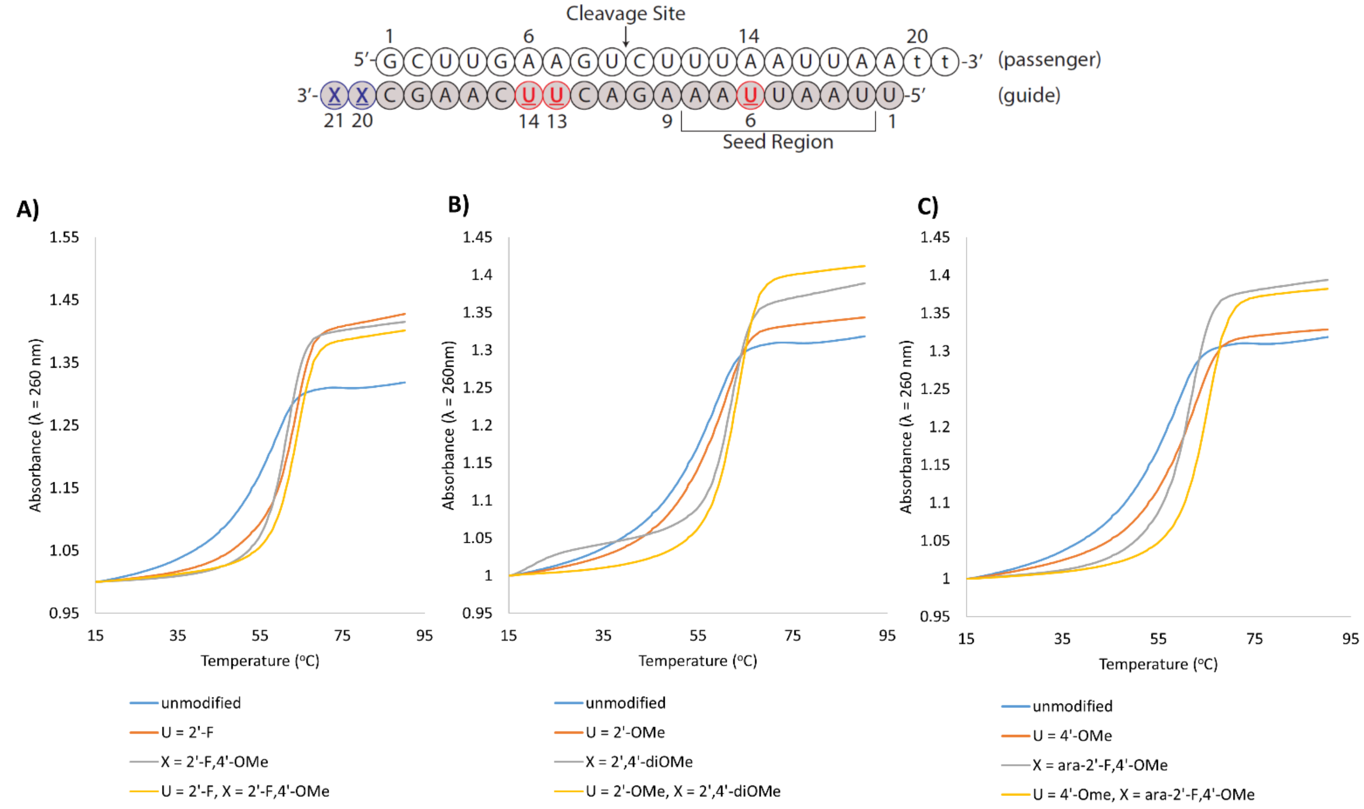

Figure S3. UV thermal melting of siRNA duplexes comparing modifications within the guide strand when modified at positions 6, 13, and 14; modified at positions 20 and 21; and modified at positions 6, 13, 14, 20 and 21. Panel **A)** shows the results of the comparisons with 2’-F ribose and 2’-F,4’-OMe; panel **B)** shows the results of the comparisons for 2’-OMe and 2’,4’-diOMe; and panel **C)** shows the results of the comparisons with 4’-OMe and 2’-F,4’-OMe arabinose.

S2. Mass Analysis of Oligonucleotides

Table S1. HRMS analysis of oligonucleotides synthesised in this study.[a]

Strand	Modification	Sequence 5'-3'[b]	Expected mass	Obtained mass
**2F-3**	2'-F	UUA AU**U** AAA GAC **UU**C AAG Cgg	6675.9	6676.0
**2F4O-3**	2'-F,4'-OMe-rU	UUA AU**U** AAA GAC **UU**C AAG Cgg	6765.9	6766.0
**2OM-3**	2'-OMe-rU	UUA AU**U** AAA GAC **UU**C AAG Cgg	6712.0	6711.8
**diOM-3**	2',4'-diOMe-rU	UUA AU**U** AAA GAC **UU**C AAG Cgg	6802.0	6802.1
**96-1**	4'-OMe-dT	UUA AU**T** AAA GAC **TT**C AAG Cgg	6754.0	6754.1
**91-1**	2'-OMe,4'-F-rU	UUA AU**U** AAA GAC **UU**C AAG Cgg	6765.9	6766.0
**91-2**	2'-F,4'-OMe-araU	UUA AU**U** AAA GAC **UU**C AAG Cgg	6765.9	6765.8
**59-3**	2'-F-araU	UUA AU**U** AAA GAC **UU**C AAG Cgg	6675.9	6675.8
**AS**	Unmodified antisense strand	UUA AUU AAA GAC UUC AAG Cgg	6669.9	6670.0
**73-1**	Unmodified sense strand	GCU UGA AGU CUU UAA UUA Att	6613.9	6613.9
**75-1**	2'-F,4'-OMe-rU	UUA AUU AAA GAC UUC AAG C**UU**	6687.9	6688.0
**75-2**	2'-F,4'-OMe-araU	UUA AUU AAA GAC UUC AAG C**UU**	6687.9	6688.0
**96-2**	4'-OMe-dT	UUA AUU AAA GAC UUC AAG C**UU**	6679.9	6680.0
**98-1**	2'-OMe-rU	UUA AUU AAA GAC UUC AAG C**UU**	6651.9	6651.9
**98-2**	2'-F-araU	UUA AUU AAA GAC UUC AAG C**UU**	6627.9	6627.9
**78-1**	2'-F-rU	UUA AUU AAA GAC UUC AAG C**UU**	6627.9	6627.8
**76-1**	2',4'-diOMe-rU	UUA AUU AAA GAC UUC AAG C**UU**	6711.9	6712.0
**95-1**	rU	UUA AUU AAA GAC UUC AAG C**UU**	6623.9	6623.9
**103-1**	2'-OMe,4'-F-rU	UUA AUU AAA GAC UUC AAG C**UU**	6687.9	6687.9
**101-1**	**U** = 2'-F-rU<br>**X** =2'-F,4'-OMe-rU	UUA AU**U** AAA GAC **UU**C AAG C**XX**	6693.9	6694.0
**101-2**	**U** = 2'-OMe-rU<br>**X** =2',4'-diOMe-rU	UUA AU**U** AAA GAC **UU**C AAG C**XX**	6754.0	6754.1
**102-1**	**U** =4'-OMe-dT<br>**X** = 2'-F,4'-OMe-araU	UUA AU**U** AAA GAC **UU**C AAG C**XX**	6771.9	6772.1
**112-1**	**U** = 2'-F-rU<br>**X** =2'-F,4'-OMe-rU	UUC U**U**G AUG AGC **U**GG **U**UC C**XX**	6671.8	6672.3
**112-2**	**U** = 2'-F-rU<br>**X** =2'-F,4'-OMe-rU	UUC UUG AUG AGC UGG UUC C**XX**	6665.8	6666.3
**93-2**	Unmodified antisense negative control	GGA ACC AGC UCA UCA AGA AUU	6700.0	6700.0
**93-1**	Unmodified sense negative control	UUC UUG AUG AGC UGG UUC CUU	6601.8	6601.9

[a]All oligonucleotides were examined by ESI-MS using a Thermo Scientific Exactive Plus Orbitrap mass spectrometer or a Bruker Daltonics Maxis Impact quadrupole time-of-flight (QTOF) mass spectrometer

[b]All nucleotides are ribonucleotides with the exception of: lowercase letters = deoxyribonucleotides.

U, **T**, and **X** are positions of modified nucleotides. **U** is uridine, **T** is Thymine and **X** is to distinguish from multiple modifications in the same strand and is defined in the second column

S3. 4'-OMe-dT P31 NMR Spectrum

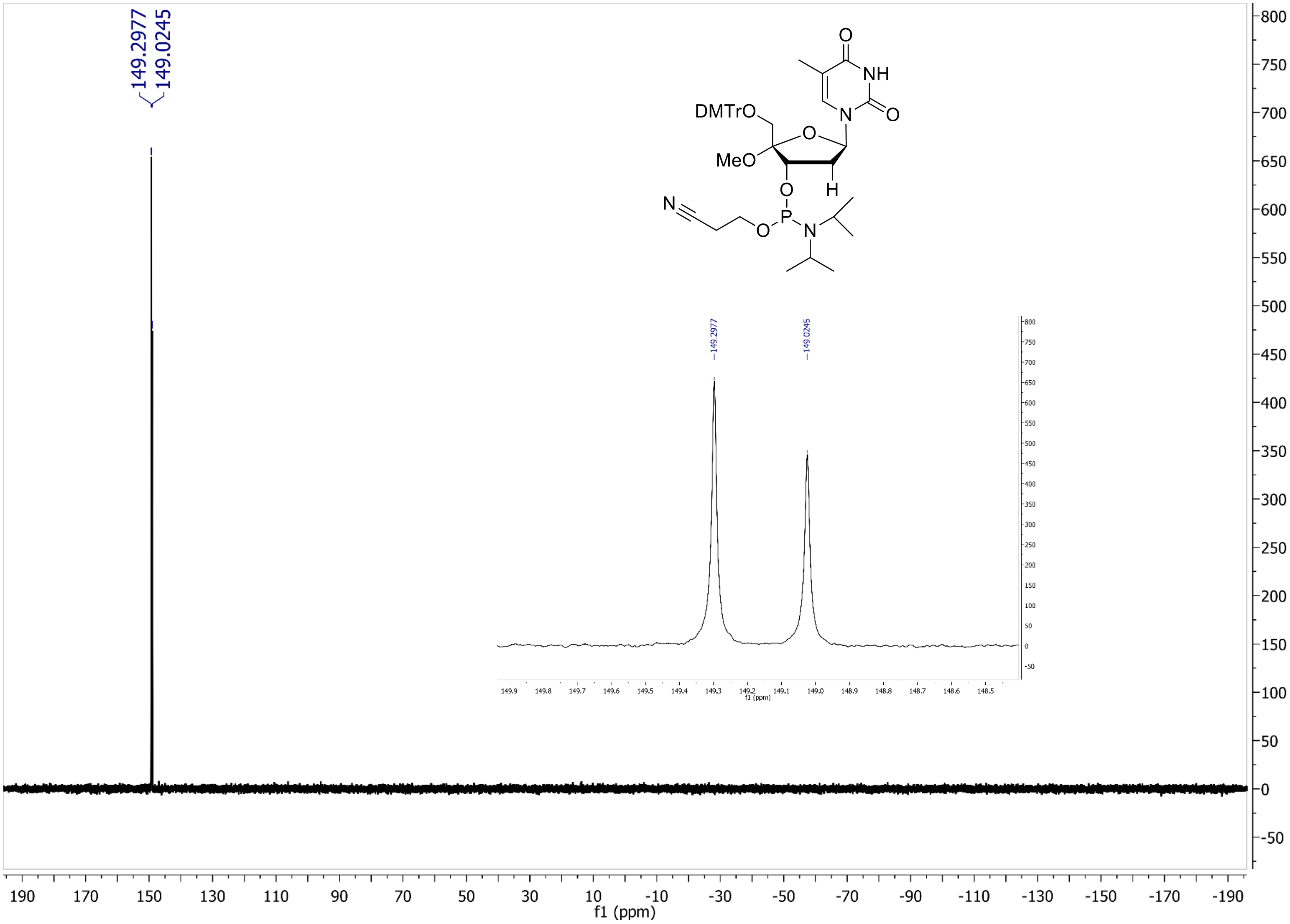

^{31}P NMR (500MHz, CD_3CN, 298K), HRMS calc. for $C_{41}H_{51}O_9N_4NaP$ $[M + Na]^+$ 797.3286, found 797.3289.
